# Sensory integration processes characterize concussed athletes with balance deficits

**DOI:** 10.1162/IMAG.a.50

**Published:** 2025-06-20

**Authors:** Bhagyashree Singh, Ingo Helmich

**Affiliations:** Department of Motor Behavior in Sports, Institute of Health Promotion and Clinical Movement Science, German Sport University Cologne, Cologne, Germany; Department of Exercise and Sport Studies, Smith College, Northampton, MA, United States

**Keywords:** sport-related concussions (SRC), mild traumatic brain injury (mTBI), functional near-infrared spectroscopy (fNIRS), post-concussion symptoms (PCS), sensory integration, postural control

## Abstract

Impaired postural control constitutes a major symptom after mild traumatic brain injuries (mTBI/sport-related concussions (SRC)). In order to uphold cognition and behavior during pathological situations, individuals may be characterized by neuronal upregulation. Because postural control necessitates the integration of sensory information within somatosensory (/parietal) cortices, we investigated the hypothesis that athletes with ongoing symptoms after SRC are characterized by increased brain activation within these areas in order to compensate for postural deficits. Sixty-six athletes (27 ± 13 years; 50 men, 16 women) participated in the study. Twenty-two concussed athletes reported high post-concussion symptoms (PCS; symptomatic group), and 22 concussed athletes reported low PCS (asymptomatic group). Twenty-two healthy non-concussed athletes served as a control group. Postural control was assessed by a pressure distribution measuring plate during four balance conditions with eyes closed/open whilst either standing on a stable/unstable surface. Brain oxygenation was collected during postural control tasks by functional near-infrared spectroscopy (fNIRS) above pre- and postcentral cortices of both hemispheres. Increased postural sway was found in symptomatic athletes when compared to control athletes’ overall conditions as well as during unstable surface conditions. Symptomatic athletes were characterized by increased brain activation within the parietal cortex overall balance conditions and when compared to asymptomatic athletes. Increased brain activation within somatosensory and parietal cortices during postural control indicates that sensory integration processes are upregulated in concussed athletes with persisting symptoms. However, such potentially compensatory processes seem to constitute an ineffective neuronal mechanism as affected athletes cannot countervail post-concussion balance deficits.

## 1 Introduction

The incidence of sport-related concussions (/mild traumatic brain injuries (mTBI)) is between 1.6 and 3.8 million annually in the USA (Langlois et al., 2006). SRC are associated to several symptoms such as headaches, dizziness, memory problems, and so forth (Didehbani et al., 2013;Echemendia et al., 2023;Lovell et al., 2006;Patricios et al., 2023) that can be long-lasting. In fact, repetitive head impacts have been associated to neurodegeneration in American football players (Daneshvar et al., 2021;Lehman et al., 2012;Nguyen et al., 2019) and soccer players (Mackay et al., 2019;Ueda et al., 2023).

There is a growing body of evidence that athletes with SRC have double the odds of sustaining a musculoskeletal injury after they return to sport compared to athletes without SRC (Brooks et al., 2016;Fino et al., 2019;Herman et al., 2017;Howell et al., 2018;Lynall et al., 2015).Howell et al. (2018)investigated dual-task gait patterns and symptoms in athletes post-concussion. Athletes who went on to sustain a subsequent time-loss injury after returning to sports demonstrated significant average walking speed dual-task cost worsening across time. In contrast, athletes who did not sustain an additional injury walked with consistent dual-task cost values across time (Howell et al., 2018). This indicates that SRC affect the motor control system.

Several studies showed long-term impacts of SRC onto the neuro-motor system (De Beaumont, Brisson, et al., 2007;De Beaumont et al., 2011,^2012^;De Beaumont, Lassonde, et al., 2007;Howell, Buckley, et al., 2018;Howell, Lynall, et al., 2018;Powers et al., 2014). Previously concussed athletes showed altered postural control, and increased intracortical inhibition within the motor cortex that was related to the number of previous concussions (De Beaumont et al., 2011). It has, therefore, been argued that SRC particularly affect the sensorimotor system (Chmielewski et al., 2021), which results in an increased injury risk (McPherson et al., 2019), decreased neuromechanical responsiveness (Wilkerson et al., 2017), altered perception-action coupling (Eagle et al., 2020), and/or subtle neurocognitive and neuromuscular deficits (Howell, Lynall, et al., 2018).

Postural control dysfunction constitutes a core symptom of mTBI (Al-Husseini et al., 2022;Buckley et al., 2021;Cavanaugh et al., 2005;Felipe, 2021;Gera et al., 2018;Guskiewicz, 2011;Guskiewicz et al., 1997,^2001^;Helmich et al., 2016,^2020^;Jacob et al., 2022;Johnston et al., 2017;Kleffelgaard et al., 2012;Kumai et al., 2022;Lin et al., 2015;Manley et al., 2017;Martini et al., 2023;McCrory et al., 2017;Peterka, 2018;Purkayastha et al., 2019;Schmidt et al., 2018;Slobounov et al., 2005;Sosnoff et al., 2011;Valovich McLeod & Hale, 2015;Wood et al., 2019). Thus far, balance problems showed to be present 30% of the time following a concussive injury (Guskiewicz, 2011), which can remain for weeks or even years after the incident (Broglio & Puetz, 2008;Ingersoll & Armstrong, 1992;Sosnoff et al., 2011;Thompson et al., 2005;Valovich McLeod & Hale, 2015;Wood et al., 2019). Concussed individuals showed to be highly affected from postural deficits when sensory input is being altered such as during eyes closed conditions, and/or unstable surface conditions (Gera et al., 2018;Guskiewicz, 2011;Guskiewicz et al., 2001;Helmich et al., 2016,^2020^;Lin et al., 2015;Martini et al., 2023). When controlling posture, sensory information must be integrated into the motor-sensory system (Horak, 2006;Peterka, 2002,^2018^;Solis-Escalante et al., 2019;Van Der Kooij & Peterka, 2011). Decreased balance function has been attributed to deficits of sensory integration processes (Gera et al., 2018;Guskiewicz, 2011;Guskiewicz et al., 1997); however, this assumption has not been assessed via brain imaging tools yet. In fact, common neuroimaging methods such as Magnetic Resonance Imaging (MRI) and/or Positron Emission Tomography (PET) are restricted in the recording of real-life scenarios such as postural control tasks (Stangl et al., 2023).

Thus far, only a few studies investigated neuronal processes during postural control tasks in individuals with mTBI by the application of functional near-infrared spectroscopy (fNIRS) and/or Electroencephalography (EEG;De Beaumont et al., 2011;Helmich et al., 2016,^2020^;Jacob et al., 2022;Slobounov et al., 2005;Thompson et al., 2005;Urban et al., 2020). The investigation of movement-related cortical potentials (MRCP) measured by EEG in concussed athletes revealed a persistent reduction of MRCP amplitude in frontal and posterior brain regions prior to initiation of postural movement up to 30 days post-injury (Slobounov et al., 2005). This study also showed that brain activation patterns were altered although abnormal postural responses recovered within 10 days post-injury (Slobounov et al., 2005). The investigation of EEG power spectral density in delta and theta bands revealed that a symptomatic concussion group could be differentiated from a non-concussed group when applying a combined setup of balance and neuroimaging methods (Jacob et al., 2022). The authors concluded that concussed individuals may increase attention and cognitive effort when compared to individuals without a history of concussion (Jacob et al., 2022). Further studies indicated that athletes with SRC are characterized by hyperactivation in parietal cortices during postural control tasks (Urban et al., 2020). When balancing on an unstable (/foam) surface with open eyes, concussed individuals demonstrated hyperactivation in the left inferior parietal cortex (IPC) as well as in the left DLPFC but lower activation the right-hemispheric parietal cortex (Urban et al., 2020).

The parietal cortex may constitute a central interface in which visual, cognitive, and motor-related signals are integrated in order to influence task-dependent motor control (Freedman & Ibos, 2018). When individuals receive tactile stimuli on their feet and are asked to locate the input, sensory processes are coded within the parietal cortex (Klautke et al., 2023). Because concussed participants showed to respond more strongly to visual and vestibular stimuli during upright stance, it has been argued that SRC may increase the dependence on visual and vestibular feedback (Caccese et al., 2021). This indicates that concussed individuals may be particular depended on the increased integration of sensory information within somatosensory (/parietal) cortices during the control of posture. Furthermore, improvement in postural control following rehabilitation treatment in individuals’ acquired brain injuries is associated to reduced functional connectivity in sensorimotor cortices (Joubran et al., 2022). This further points out that SRC may increase sensory integration processes during the control of posture. With regard to previously observed parietal hyperactivation during postural control (Urban et al., 2020) and the argument that concussed individuals may increase their cognitive effort to control balance (Jacob et al., 2022), we hypothesized that athletes with persistent symptoms after SRC are characterized by neural upregulation in the postcentral (sensory/parietal) cortex during sensory integration processes, that is, when controlling posture during altered sensory balance conditions.

## 2 Methods

### 2.1 Participants

Sixty-six individuals (mean age: 23.4 ± 5.2 years (minimum [min]: 16, maximum [max]: 40; 50 men, 16 women) participated in this study. A priori power analysis by using G*Power 3.1.9.7 indicated that 36 participants were necessary for a statistical analysis between groups and repeated measures (effect size f = 0.25, Power = 0.95, calculated critical F = 2.696, calculated actual Power = 0.95). All participants were active athletes from various sports, including American football, soccer, and ice hockey. Individuals were categorized into three groups based on their post-concussion symptom scale score and their concussion history: 22 concussed and symptomatic athletes (PCSS > 10:*symptomatic*group (PCSS = 26.9 ± 14.3 (min: 12, max: 61)); mean age: 24.4 ± 5.7 years (min: 16, max: 39); number of experienced concussions: 2.8 ± 1.9 years (min: 1, max: 7)), 22 concussed and asymptomatic athletes (PCSS ≤ 10:*asymptomatic*group (PCSS = 3.3 ± 2.9 (min: 0, max: 10)); mean age: 24.05 ± 5.3 years (min: 16, max: 40); number of experienced concussions: 2.0 ± 1.6 years (min: 1, max: 8)), and 22 non-concussed and asymptomatic athletes (PCSS ≤ 10:*control*group (PCSS = 2.0 ± 2.9 (min: 0, max: 0); mean age: 21.8 ± 4.2 years (min: 16, max: 30),Table 1). Thus, groups differed significantly regarding their PCSS scores (*PCSS*, F(2, 63) = 58.780, p < 0.001)). The two concussed groups also significantly differed regarding their time post the concussive incident (t(42) = -2.392, p < 0.05). The time post injury was on average shorter in the symptomatic group (mean time post-concussion: 14.8 ± 5.5 months) when compared to the asymptomatic group (mean time post-concussion: 33.3 ± 5.5 months;Table 1). Furthermore, the groups differed regarding their time of participation in sports (F(2, 63) = 4.096, p < 0.05). The symptomatic showed to participate in sports significantly longer than the asymptomatic group (p < 0.05). The symptomatic and asymptomatic concussed groups did not significantly differ regarding the number of experienced concussions (χ^2^(df = 6, N = 44) = 7.974, p = 0.240). Asymptomatic and control athletes were matched to the symptomatic group for age and gender (i.e., no significant differences were found between groups (*age*, χ^2^(df = 38, N = 66) = 29.871, p = 0.824;*gender*, χ^2^(df = 2, N = 66) = 4.620, p = 0.099)).

**Table 1.** Participants.

	Symptomatic Athletes	Asymptomatic Athletes	Control Athletes
Number of participants	22	22	22
Gender (female/male)	8/14	6/16	2/20
Age (years)	24.4 ± 5.7	24.1 ± 5.3	21.8 ± 4.2
PCSS score *	26.9 ± 14.3	3.3 ± 2.9	2.0 ± 2.9
Experienced concussions	2.8 ± 1.9	2.0 ± 1.6	-
Time post-concussion (months) *	14.8 ± 5.5	33.3 ± 5.5	-
Years of sport participation *	13.1 ± 5.4	8.9 ± 4.2	9.9 ± 5.4

* Indicates significant differences between groups.

PCSS: Post-concussion symptom scale score.

Exclusion criteria included a history of neurological, psychiatric, equilibrium, or hearing disorders that could potentially interfere with their ability to perform balance and cognitive tasks. Prior to participation, all participants provided informed consent after being instructed about the experimental procedures. The study was approved by the local Ethics Committee.

### 2.2 Posturography

**Fig. 1.**
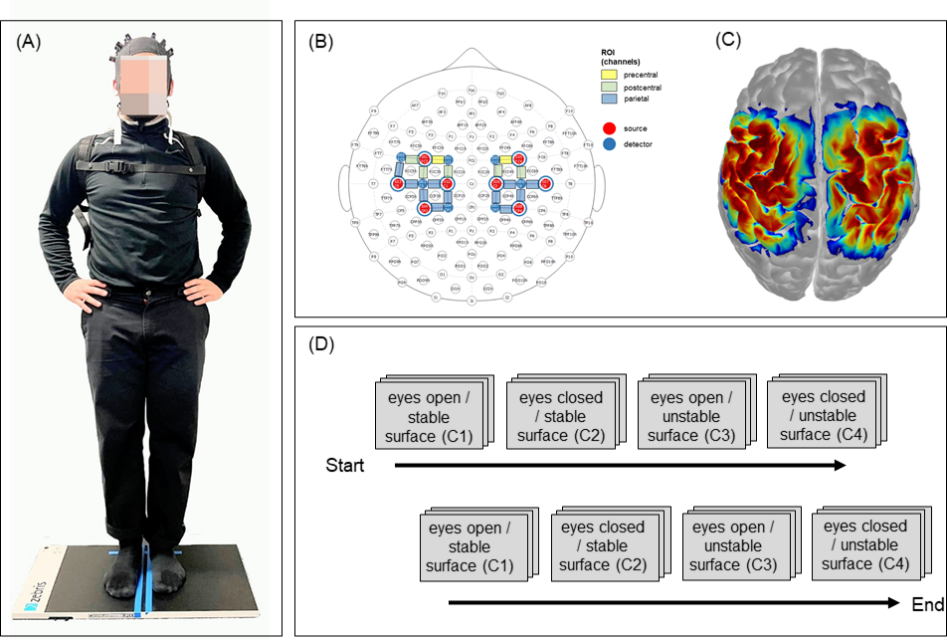
(A) Exemplary participant and experimental setup. (B) fNIRS optode placement according to the 10–20-system (with three regions of interest (ROI): precentral, postcentral, parietal. (C) fNIRS sensitivity map. (D) Study design (six trials per condition separated in two blocks (each trial lasted 10 seconds)).

Individuals were instructed to control their posture in a standardized standing position (distance between feet: 2 cm, hands on hips, view straight ahead on a marked cross;Fig. 1). Four postural control conditions were carried out according to the Clinical Test of Sensory Interaction in Balance (Shumway-Cook & Horak, 1986), which examines combinations of visual and tactile manipulations during balance control. However, we modified conditions in order to make balance conditions compatible to brain activation measures applying fNIRS. Thus, postural control tasks were either performed with open and/or closed eyes and/or either on a firm (/stable) surface or on an unstable surface: condition 1 (c1): eyes open and stable surface; condition 2 (c2): eyes closed and stable surface; condition 3 (c3): eyes open and unstable surface; condition 4 (c4): eyes closed and unstable surface. The unstable surface was created using a piece of six cm thick foam pad (“AIREX Balance-Pad”). We created a block design with two blocks of each balance condition. Each block included three trials (10 seconds per trial; 15 seconds between conditions/instruction; 5 seconds between trials), resulting in a total of six trials per condition (Fig. 1). Between conditions, the participants were asked to stand still with their hands on their hips and open eyes. We applied a force plate system (“ZEBRIS platform, type FDM-S”, measure frequency 240 Hz) to register center of mass displacement (/postural sway) by measuring ground reaction forces. This system provides information about the ability to keep postural control by the registration of the deviations from the center of pressure (COP) by the mean length of the movement path (/track) per time (millimeters/second [mm/s]); COP track length is defined as the absolute length of the COP path movements throughout the testing period (10 seconds). The second parameter COP area (measured in square millimeters [mm2]) provides information about the area that is used to control posture during the 10 seconds of sway. The means of COP track length and COP area were exported for each subject and each condition for statistical analyses.

### 2.3 fNIRS acquisition and analysis

Functional near-infrared spectroscopy (fNIRS) maps human brain function by measuring and imaging local changes in hemoglobin concentrations in the brain that arise from the modulation of cerebral blood flow and oxygen metabolism by neural activity (Yücel et al., 2017). Similar to functional magnetic resonance imaging (fMRI), fNIRS detects neurovascular coupling to infer changes in neuronal activity. Neurovascular coupling refers to changes in local neural activity, which are correlated to changes in local cerebral blood flow (CBF;Tachtsidis & Scholkmann, 2016). Cerebral oxygenation changes were recorded using a portable continuous wave fNIRS system (NIRSport 2, NIRx, Medical Technologies LLC, Berlin, Germany; wavelengths of 760 and 850 nm; sampling rate 10.2 Hz). The NIRSport 2 system contains eight light sources and eight light detectors, and a short-distance detector bundle. The optodes were placed according to the 10–20-system (Jasper, 1958) above pre- and postcentral gyri of each hemisphere (Fig. 1;Table 2) using a standardized cap (EasyCap GmbH, Herrsching, Germany) and registrated via the fNIRS Optodes’ Location Decider (fOLD) toolbox (Zimeo Morais et al., 2018). The optodes covered pre- and postcentral cortices, including the primary motor cortex, primary somatosensory cortex, and parietal sensory association cortex in both the right and left hemisphere of the brain (Fig. 1;Table 2). Data were recorded from 18 (long-distance) channels of measurement. Eight short-distance (8 mm) channels were additionally applied to account for changes in extra-cerebral blood flow. The mean source-detector distance (long-distance channels) was 38.1 ± 2.7 mm.

**Table 2.** fNIRS channel locations and regions of interest (ROI).

Channel	10–20-system	MNI coordinates	Landmarks	Hemisphere	ROI
S1-D1/Ch1	FC3-FC1	-38 12 55	Precentral Gyrus	LH	precentral
S5-D5/Ch11	FC4-C4	44 25 40	Precentral Gyrus	RH	precentral
S1-D2/Ch2	FC3-FC5	-55 12 34	Postcentral Gyrus	LH	postcentral
S1-D3/Ch3	FC3-C3	-50 -3 50	Postcentral Gyrus / Superior parietal lobe	LH	postcentral
S2-D1/Ch4	C1-FC1	-26 -5 68	Postcentral Gyrus	LH	postcentral
S5-D6/Ch12	FC4-C4	52 -4 48	Postcentral Gyrus	RH	postcentral
S6-D5/Ch13	C2-FC2	27 -4 68	Postcentral gyrus/ Superior parietal lobe	RH	postcentral
S2-D3/Ch5	C1-C3	-42 -20 -62	Supramarginal Gyrus posterior division	LH	parietal
S2-D4/Ch6	FC3-C3	-50 -3 50	Superior parietal lobe	LH	parietal
S3-D2/Ch7	C5-FC5	-62 -3 23	Supramarginal gyrus anterior division	LH	parietal
S3-D3/Ch8	C5-C3	-60 -18 37	Supramarginal gyrus posterior division	LH	parietal
S4-D3/Ch9	CP3-C3	-52 -34 52	Superior parietal lobe	LH	parietal
S4-D4/Ch10	CP3-CP1	-39 -48 60	Superior parietal lobe	LH	parietal
S6-D6/Ch14	C2-C4	42 -21 62	Superior parietal lobe, right supramarginal Gyrus	RH	parietal
S6-D7/Ch15	C2-CP2	28 -36 71	Superior parietal lobe	RH	parietal
S7-D6/Ch16	C6-C4	62 -20 37	Supramarginal Gyrus posterior division, Angular Gyrus	RH	parietal
S8-D6/Ch17	CP4-C4	53 -35 52	Superior parietal lobe, Angular Gyrus	RH	parietal
S8-D7/Ch18	CP4-CP2	39 -49 60	Superior parietal lobe	RH	parietal

The fNIRS data were analyzed using the Satori (v.1.8) toolbox (Lührs & Goebel, 2017). The raw intensity data were converted to optical density, which was converted via the modified Beer–Lambert law (MBLL) into concentration changes of oxygenated hemoglobin (∆HbO_2_) and deoxygenated hemoglobin (∆HbR). Triggers were set manually for each trial of postural control tasks. Movement artifacts were corrected by applying the (I) spike removal motion correction function (settings: iterations: 10, lag: 5 seconds, threshold: 3.5, influence: 0.5, interpolation: monotonic) and (II) the temporal derivative distribution repair (TDDR) function according toFishburn et al. (2019). Because the use of short-separation detector measurements as a regressor in the general linear model (GLM) has been previously shown to statistically improve HRF estimation (Gagnon et al., 2011;Tachtsidis & Scholkmann, 2016), we used short-distance signals to regress out signals from extra-cerebral layers from the long-distance channels. To account for cardiac oscillations and Mayer-waves, we used a 0.4 Hz low-pass filter, a high-pass filter (butterworth) of 0.01 Hz, and the linear detrending function of Satori. The data were normalized by z-transformation, and the betas of the hemodynamic response were estimated applying the GLM. Betas of each channel and condition were exported for further statistical analyses.

### 2.4 Statistics

**Appendix Fig. A1.**
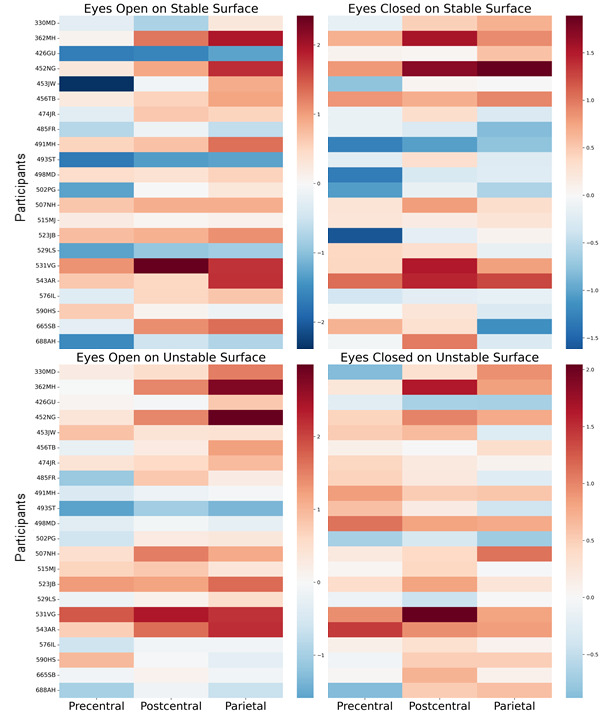
Individual brain oxygenation variation maps (∆HbO_2_) of concussed and symptomatic versus control athletes within precentral, postcentral, and parietal brain regions during (4) postural control conditions (with eyes open/closed and during stable/unstable surface conditions).

Comparisons of the mean(s) (repeated (rmANOVA) analyses of variance) were performed for postural control (COP area, COP track length) and brain oxygenation parameters (∆HbO_2_, ∆HbR) using IBM SPSS statistics (Version 28). Each parameter was additionally tested by univariate analyses (uniANOVA). For the betas of ∆HbO_2_and ∆HbR as well as for COP area and COP track length, we calculated the repeated within-subjects factors*vision*(eyes opened/closed) and*surface*(stable/unstable surface). For the analysis of brain activity, we additionally integrated the within-subjects factor*regions of interest (ROI)*: (I) precentral, (II) postcentral, and (III) parietal cortices. The three*ROIs*were calculated by the mean oxygenation of the channels covering the corresponding brain regions: precentral: ch1 and ch11; postcentral: ch2–ch4, and ch12–ch13; parietal: ch5–ch10, and ch14–ch18 (Table 2). The between-subject factor*group*was calculated between*symptomatic*,*asymptomatic*and*control*individuals. Significant results are reported from p < 0.05. Multiple post hoc pairwise comparisons were corrected with Bonferroni corrections. If the requirements of the ANOVA (i.e., sphericity) were violated, we used the Greenhouse-Geisser correction. Furthermore, t-contrasts of brain activation during postural control were calculated applying the GLM (with the threshold of p < 0.05). The following contrasts were statistically calculated for each group separately: (I) eyes closed versus eyes open condition overall*surface*conditions; (II) unstable surface versus stable surface condition overall*vision*conditions; and (III) overall conditions versus baseline. The baseline refers to any period not defined as a task, that is, here this concerned standing with hands resting on the hips and eyes opened. Visual depiction of t-contrasts (∆HbO_2_) was performed for each channel by applying the Satori toolbox. Furthermore, in order to understand the individual variation of concussed and symptomatic athletes from both other groups, we added visual variation maps (∆HbO_2_) for each condition to theAppendix Figures A1andA2. According to best practice suggestions (Yücel et al., 2021), we report significant effects of both chromophores (∆HbO_2_, ∆HbR) in the results section. However, in line with previous studies (e.g.,Helmich et al., 2020;Hocke et al., 2018;Urban et al., 2020), we focus on ∆HbO_2_in the discussion.

## 3 Results

### 3.1 Postural control

#### 3.1.1 Between group effects

**Fig. 2.**
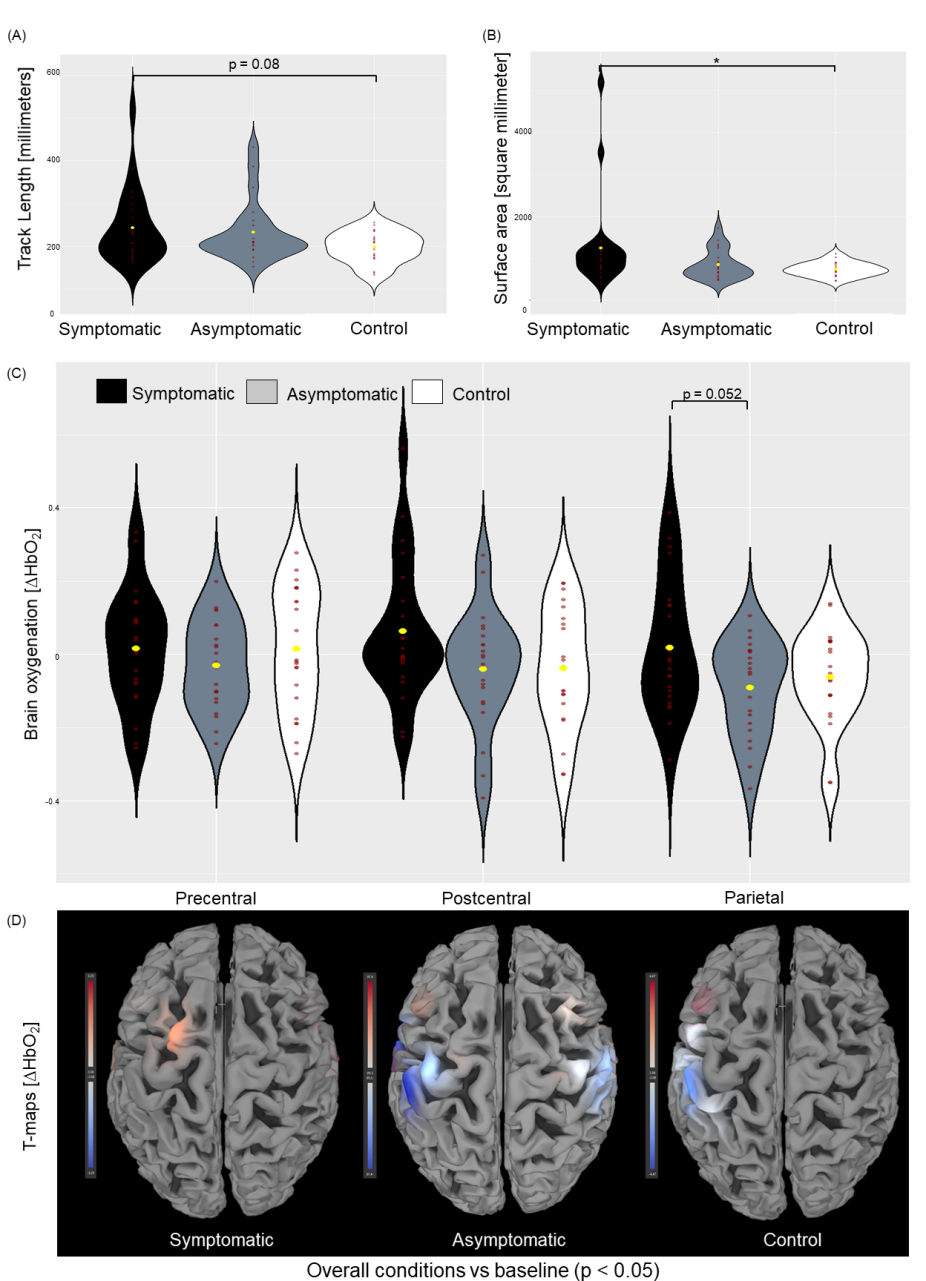
Postural control ((A) track length; (B) surface area) and brain activation ((C) beta values of ∆HbO_2_within precentral, postcentral, and parietal regions of interest (ROI); (D) t-values all conditions vs. baseline (threshold: p < 0.05)) between symptomatic, asymptomatic, and control athletes overall balance conditions (* indicates p < 0.05).

The rmANOVA showed (over both balance parameters) a significant effect of*group*(F(4, 126) = 2.888, p < 0.05, partial eta squared [η^2^] = 0.084;Table 3). The univariate ANOVA (uniANOVA) for the factor COP area revealed a statistically significant effect for the factor*group*(F(2, 63) = 3.654, p < 0.05, η^2^= 0.104). Post-hoc pairwise comparisons showed a significantly increased COP area in the*symptomatic*group when compared to the*control*group (p < 0.05;Fig. 2B).

**Table 3.** Statistical (significant) results for postural control (COP (center of pressure) area, COP track length).

Factor	df	F	Partial η ^2^	p-value	Post-hoc pairwise comparison
**Between-group effects**					
**Group**	4, 126	2.888	0.084	<0.05	
COP area	2, 63	3.654	0.104	<0.05	symptomatic > control (p < 0.05)
**Group × Vision**	4, 126	3.305	0.095	<0.05	
COP area	2, 63	3.178	0.092	<0.05	closed eyes: symptomatic > control (p < 0.05)
**Within-group effects**					
**Vision**	2, 62	316.896	0.911	<0.001	
COP area	1, 63	67.008	0.515	<0.001	eyes closed > open (p < 0.001)
COP track length	1, 63	214.528	0.773	<0.001	eyes closed > open (p < 0.001)
**Surface**	2, 62	112.783	0.784	<0.001	
COP area	1, 63	264.386	0.808	<0.001	unstable > stable (p < 0.001)
COP track length	1, 63	639.551	0.910	<0.001	unstable > stable (p < 0.001)
**Vision × Surface**	2, 62	92.729	0.749	<0.001	
COP area	1, 63	109.735	0.635	<0.001	eyes open: unstable > stable (p < 0.01)
					eyes closed: unstable > stable (p < 0.001)
					stable: eyes closed > open (p < 0.001)
					unstable: eyes closed > open (p < 0.001)
COP track length	1, 63	188.248	0.749	<0.001	eyes open: unstable > stable (p < 0.001)
					eyes closed: unstable > stable (p < 0.001)
					stable: eyes closed > open (p < 0.001)
					unstable: eyes closed > open (p < 0.001)

The rmANOVA showed over both parameters a significant effect of*group × vision*(F(4, 126) = 3.305, p < 0.05, η^2^= 0.095). The uniANOVA for the factor COP area demonstrated a statistically significant interaction effect for*group × vision*(F(2, 63) = 3.178, p < 0.05, η^2^= 0.092;Table 3). Post-hoc comparisons revealed a significantly increased COP area in the*symptomatic*group as compared to the*control*group during the closed eyes condition (p < 0.05).

#### 3.1.2 Within group effects

The rmANOVA showed a statistically significant effect for the factors*vision*(F(2, 62) = 316.896, p < 0.001, η^2^= 0.911),*surface*(F(2, 62) = 112.783, p < 0.001, η^2^= 0.784), and the interaction*vision × surface*(F(2, 62) = 92.729, p < 0.001, η^2^= 0.749;Table 3).

**Fig. 3.**
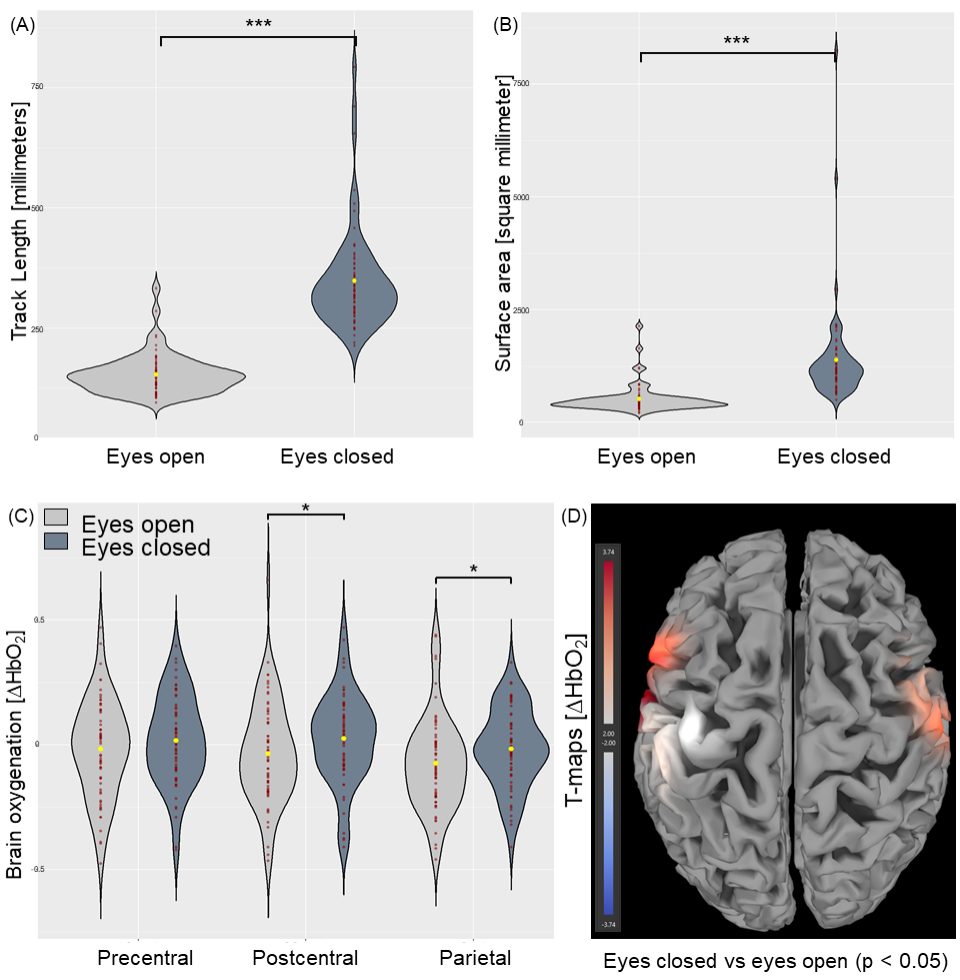
Postural control ((A) track length; (B) surface area) and brain activation ((C) beta values of ∆HbO_2_within precentral, postcentral, and parietal regions of interest (ROI); (D) t-values (threshold: p < 0.05)) overall athletes during eyes closed versus eyes open conditions (* indicates p < 0.05; *** indicates p < 0.001).

**Fig. 4.**
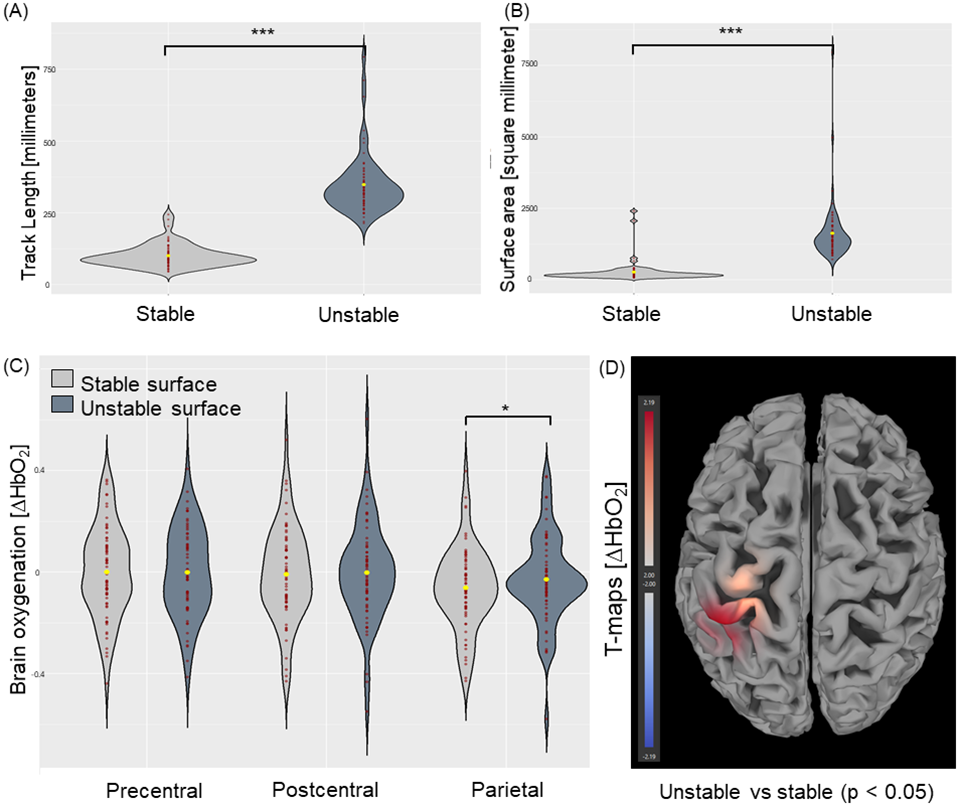
Postural control ((A) track length; (B) surface area) and brain activation ((C) beta values of ∆HbO_2_within precentral, postcentral, and parietal regions of interest (ROI); (D) t-values (threshold: p < 0.05)) overall athletes during unstable versus stable surface conditions (* indicates p < 0.05; *** indicates p < 0.001).

The uniANOVA for COP area showed a statistically significant effect for the factor*vision*(F(1, 63) = 67.008, p < 0.001, η^2^= 0.515),*surface*(F(1, 63) = 264.386, p < 0.001, η^2^= 0.808), the interaction*vision × surface*(F(2, 63) = 109.735, p < 0.001, η^2^= 0.635;Table 3). Post-hoc comparisons for the factor*vision*demonstrated an increased COP area during the eyes closed condition when compared to the eyes open condition (p < 0.001;Fig. 3). Post-hoc comparison for factor*surface*demonstrated an increased COP area during the unstable surface condition when compared to the stable surface condition (p < 0.001;Fig. 4). Post-hoc comparison for the interaction of*vision × surface*showed an increased COP area during the open eyes condition on the unstable surface when compared to the stable surface condition (p < 0.01); during the closed eyes condition on an unstable surface when compared to the stable surface condition (p < 0.001); during the stable surface condition with closed eyes when compared to the open eyes condition (p < 0.001); and during the unstable surface with closed eyes condition and when compared to the open eyes condition (p < 0.001;Table 3).

The uniANOVA for COP track length showed significant effects for the within-subjects factors*vision*(F(1, 63) = 214.528, p < 0.001, η^2^= 0.773) and*surface*(F(1, 63) = 639.551, p < 0.001, η^2^= 0.910), and for the interaction of*vision × surface*(F(1, 63) = 188.248, p < 0.001, η^2^= 0.749;Table 3). Post-hoc comparison for factor*vision*demonstrated an increased COP track length during the eyes closed condition when compared to the eyes open condition (p < 0.001;Fig. 3A). Post-hoc comparison for the factor*surface*demonstrated an increased COP track length during the unstable surface condition when compared to the stable surface condition (p < 0.001). Post-hoc comparison for the interaction of*vision × surface*showed in increased COP track length during the open eyes condition on unstable surface when compared to the stable surface condition (p < 0.001); during the closed eyes condition on an unstable surface when compared to the stable surface condition (p < 0.001); during the stable surface condition with closed eyes when compared to the open eyes condition (p < 0.001); and during the unstable surface with closed eyes condition and when compared to the open eyes condition (p < 0.001;Table 3).

### 3.2 Brain activation

#### 3.2.1 ANOVAs of the betas

##### 3.2.1.1 Between group effects

The uniANOVA for ΔHbO_2_showed significant*group*effects in the parietal ROI (F(2, 63) = 3.212, p < 0.05, η^2^= 0.093);Table 4). Post-hoc pairwise comparison revealed marginally significant higher ΔHbO_2_in the*symptomatic*group when compared to the*asymptomatic*group in the parietal ROI (p = 0.052;Fig. 2C).

**Table 4.** Statistical (significant) results for brain oxygenation (∆HbO_2_, ∆HbR) between (*symptomatic*,*asymptomatic*,*control*athletes) and within groups.

Factor	df	F	Partial η ^2^	p-value	Post-hoc pairwise comparison
**Between groups**					
** ∆HbO _2_**					
**Group**					
parietal	2, 63	3.212	0.093	<0.05	symptomatic > asymptomatic (p = 0.052)
**∆HbR**					
**Group × Vision**					
precentral	2, 63	3.124	0.090	=0.051	Controls: closed eyes < open eyes (p < 0.05)
**Witin groups**					
** ∆HbO _2_**					
**Vision**	3, 61	3.976	0.164	<0.05	
postcentral	1, 63	10.231	0.140	<0.01	closed eyes > open eyes (p < 0.01)
parietal	1, 63	9.620	0.132	<0.01	closed eyes > open eyes (p < 0.01)
**Surface**					
parietal	1, 63	4.436	0.066	<0.05	unstable surface > stable surface (p < 0.05)
**∆HbR**					
**Surface × Vision**					
postcentral	1, 63	5.210	0.076	<0.05	Stable surface: closed eyes > open eyes (p < 0.01)

The uniANOVA for ∆HbR showed marginally significant interaction effects for*group × vision*in the precentral ROI (F(2, 63) = 3.124, p = 0.051, η^2^= 0.090;Table 4). Post-hoc pairwise comparison revealed a significantly reduced ΔHbR in the*control*group during the eyes closed condition when compared to the eyes opened condition (p < 0.05).

##### 3.2.1.2 Within group effects

The rmANOVA for the factor*vision*showed significant effects for ΔHbO_2_(F(3, 61) = 3.976, p < 0.05, η^2^= 0.164;Table 4). The uniANOVA for the factor*vision*showed significant effects for ΔHbO_2_the postcentral (F(1, 63) = 10.231, p < 0.01, η^2^= 0.140) and parietal ROI (F(1, 63) = 9.620, p < 0.01, η^2^= 0.132). Post-hoc pairwise comparison revealed significantly increased ΔHbO_2_during the eyes closed condition when compared to the eyes open condition in the postcentral (p < 0.01) and the parietal ROI (p < 0.01;Fig. 3C).

The uniANOVA for the factor*surface*showed significant effects for ΔHbO_2_in the parietal ROI (F(1, 63) = 4.436, p < 0.05, η^2^= 0.066;Table 4). Post-hoc comparisons showed higher ΔHbO_2_during the unstable surface condition when compared to stable surface conditions (p < 0.05;Fig. 4).

The uniANOVA results revealed for ΔHbR significant effects for the interaction effect of*vision × surface*in the postcentral ROI (F(1, 63) = 5.210, p < 0.05, η^2^= 0.076). Post-hoc comparison revealed reduced ΔHbR during closed eyes when compared to open eyes conditions during the stable surface condition (p < 0.01).

#### 3.2.2 GLM t-contrasts

##### 3.2.2.1 Overall postural control conditions versus baseline (differentiated between groups)

The*symptomatic*group showed significantly increased t-contrasts (ΔHbO_2_) overall balance conditions in ch2 ((t = 2.56, p < 0.05);Table 5 (I);Fig. 2D)). The*asymptomatic*group showed significantly decreased t-contrasts in ch5 (t = -2.65, p < 0.05), ch7 (t = -3.55, p < 0.05), ch9 (t = -4.98, p < 0.05), ch14 (t = -2.16, p < 0.05), ch16 (t = -2.67, p < 0.05), and ch17 (t = -2.62, p < 0.05) The*control*group showed significantly decreased t-contrasts in ch2 (t = -2.03, p < 0.05), ch3 (t = -2.17, p < 0.05), ch8 (t = -2.21, p < 0.05), ch9 (t = -3.33, p < 0.05), and ch10 (t = -2.18, p < 0.05).

**Table 5.** Significant t-contrasts (∆HbO_2_, ∆HbR) of*symptomatic*,*asymptomatic*, and*control*athletes during postural control conditions.

	Symptomatic		Asymptomatic		Control	
	t-value	p-value	t-value	p-value	t-value	p-value
**(I) All conditions vs. baseline**						
** ∆HbO _2_**						
CH 2	0.70	0.49	-0.74	0.47	**-2.03**	**0.05**
CH 3	1.06	0.30	-0.59	0.56	**-2.17**	**0.04**
CH 4	**2.56**	**0.02**	-1.94	0.07	0.13	0.90
CH 5	1.86	0.08	**-2.65**	**0.02**	-1.50	0.15
CH 7	-0.77	0.45	**-3.55**	**0.00**	-1.78	0.09
CH 8	0.64	0.53	-1.17	0.25	**-2.21**	**0.04**
CH 9	-0.29	0.77	**-4.98**	**0.00**	**-3.33**	**0.00**
CH 10	-0.40	0.69	-1.84	0.08	**-2.18**	**0.04**
CH 14	1.02	0.32	**-2.16**	**0.04**	-1.14	0.27
CH 16	-1.44	0.17	**-2.67**	**0.01**	-0.63	0.54
CH 17	0.33	0.74	**-2.62**	**0.02**	-1.85	0.08
**∆HbR**						
CH 4	**2.46**	**0.02**	1.16	0.26	0.90	0.38
CH 11	**2.22**	**0.04**	**2.22**	**0.04**	1.03	0.31
CH 13	**2.34**	**0.03**	0.88	0.39	1.98	0.06
CH 15	**2.35**	**0.03**	1.04	0.31	1.37	0.19
**(II) Unstable vs. stable surface**						
** ∆HbO _2_**						
CH 1	**2.20**	**0.04**	-0.77	0.45	-0.10	0.92
CH 5	**2.26**	**0.04**	0.26	0.80	0.54	0.59
CH 9	**2.04**	**0.05**	0.38	0.71	0.91	0.37
CH 18	**2.62**	**0.02**	0.14	0.89	0.23	0.82
**∆HbR**						
CH 7	0.20	0.85	**2.12**	**0.046**	0.24	0.81
**(III) Eyes closed vs. eyes open**						
** ∆HbO _2_**						
CH 2	1.87	0.08	0.74	0.47	**2.50**	**0.02**
CH 5	0.08	0.94	1.60	0.13	**2.11**	**0.05**
CH 7	**2.71**	**0.01**	1.08	0.29	**2.66**	**0.01**
CH 8	0.63	0.54	0.10	0.92	**3.51**	**0.00**
CH 9	0.42	0.68	0.27	0.79	**2.72**	**0.01**
CH 16	0.92	0.37	**2.18**	**0.04**	**2.38**	**0.03**
**∆HbR**						
CH 1	-2.03	0.06	1.75	0.10	**-2.79**	**0.01**
CH 4	-1.06	0.30	-1.53	0.14	**-2.54**	**0.02**

Contrasts: (I) All conditions versus baseline; (II) Unstable versus stable surface; (III) Eyes closed versus eyes open. Bold values indicate significance.

##### 3.2.2.2 Unstable versus stable postural control conditions (differentiated between groups)

The*symptomatic*group showed significantly increased t-contrasts (ΔHbO_2_) on the unstable surface condition when contrasted to stable surface condition in ch1 (t = 2.20, p < 0.05), ch5 (t = 2.26, p < 0.05), ch9 (t = 2.04, p = 0.054), and ch18 (t = 2.61, p < 0.05;Table 5 (II)). The*asymptomatic*group showed significantly increased t-contrasts during the unstable surface condition when contrasted to stable surface condition in ch2 (t = 2.249, p < 0.05). The*control*group showed no significant contrast results.

##### 3.2.2.3 Eyes closed versus eyes open (differentiated between groups)

The*symptomatic*group showed significantly increased t-contrasts (ΔHbO_2_) during the eyes closed condition when contrasted to the eyes open condition in ch7 (t = 2.71, p < 0.05;Table 5 (III)). The*asymptomatic*group showed significantly increased in ch16 (t = 2.18, p < 0.05). The*control*group showed significantly increased t-contrasts in ch2 (t = 2.50, p < 0.05), ch5 (t = 2.11, p < 0.05), ch7 (t = 2.66, p < 0.05), ch8 (t = 3.51, p < 0.05), ch9 (t = 2.72, p < 0.05), and ch16 (t = 2.38, p < 0.05).

##### 3.2.2.4 Eyes closed versus eyes open (overall participants)

The contrast of eyes closed versus eyes open releveled overall participants significant t-contrasts (ΔHbO_2_) in ch2 (t = 3.01, p < 0.05), ch7 (t = 3.74, p < 0.05), ch12 (t = 2.67, p < 0.05), and ch16 (t = 2.92, p < 0.05;Fig. 3D;Table 6). The contrast of unstable surface versus stable surface releveled overall participants’ significant t-contrasts in ch6 (t = 2.06, p < 0.05) and ch10 (t = 2.19, p < 0.05;Fig. 4D;Table 6).

**Table 6.** Significant t-contrasts (∆HbO_2_, ∆HbR) during postural control with eyes closed versus eyes open and unstable versus stable surface conditions overall participants.

	Eyes closed vs. eyes open		Unstable vs. stable surface	
	t-value	p-value	t-value	p-value
** ∆HbO _2_**
CH 2	**3.01**	**0.00**	0.79	0.43
CH 6	1.06	0.29	**2.06**	**0.04**
CH 7	**3.74**	**0.00**	0.15	0.88
CH 10	1.48	0.14	**2.19**	**0.03**
CH 12	**2.67**	**0.01**	-0.60	0.55
CH 16	**2.92**	**0.00**	1.47	0.15
**∆HbR**				
CH 4	**-2.86**	**0.01**	0.56	0.58

Bold values indicate significance.

## 4 Discussion

We present functional neuroimaging evidence of altered brain activation in a sample of concussed (with and without ongoing post-concussion symptoms) and non-concussed athletes during postural control tasks. This study investigated postural sway and simultaneous brain oxygenation patterns in the motor-sensory cortex during balance tasks with closed and opened eyes as well as during stable and unstable surface conditions. The results showed that symptomatic concussed athletes were characterized by increased postural sway when compared to athletes without experienced concussions overall postural control conditions. Symptomatic concussed athletes showed increased brain activation within postcentral/parietal cortices overall balance conditions and when compared to asymptomatic athletes. In overall participants, increased postural sway was observed during postural control with closed eyes when compared to eyes open conditions as well as during unstable surface conditions when compared to stable surface conditions. Increased postural sway during eyes closed conditions was accompanied by increased brain oxygenation within postcentral and parietal brain regions and when compared to open eyes conditions. The contrast of unstable versus stable surface conditions revealed significant increased brain activation within parietal brain cortices.

### 4.1 Postural control

The present study showed that concussed individuals with high self-reported symptoms are characterized by increased postural sway independently of balance condition, that is, when balancing with closed and open eyes as well as when balancing on unstable and stable surfaces. Balance maintenance is achieved by integration of sensory information to the motor system (Horak, 2006;Peterka, 2002,^2018^;Solis-Escalante et al., 2019;Van Der Kooij & Peterka, 2011). Because various sensory systems (somatosensory/proprioception, visual, vestibular) contribute to balance control, it has been argued that impaired postural control after mild traumatic brain injuries may be grounded in a deficit of integrating sensory information (Gera et al., 2018;Guskiewicz, 2011;Guskiewicz et al., 2001). Standing on an unstable surface alters the somatosensory and proprioceptive inputs that need to be processed and (neuronally) integrated (Gera et al., 2018). Here, postural control showed to be impaired in concussed and symptomatic athletes overall conditions as well as during the unstable surface condition. The unstable surface reduces the accuracy of the orientation information (Guskiewicz, 2003). Increased postural sway during this condition (i.e., when proprioception from the feet is eliminated) suggests that concussed individuals cannot compensate by other sensory systems for the loss of somatosensation.Gera et al. (2018)found that postural sway was higher in concussed individuals when compared to controls during several postural control conditions such as when balancing with closed and open eyes on a stable surface as well as on an unstable surface. However, whereas the latter authors investigated concussed individuals 2–3 days post-concussion, in the present study we investigated individuals that reported ongoing symptoms long-term post-concussion. In fact, balance impairments have been reported up to 4 years after a concussive injury (Geurts et al., 1996;Kleffelgaard et al., 2012). Because we also found an effect between groups overall balance conditions, we assume that individuals that suffer from post-concussion symptoms long-term must be affected from a general defect of integrating sensory information.

### 4.2 Neural processes during postural control

Central sensorimotor integration constitutes the ability to process and integrate sensory information in order to transform it into a motor output. This function constitutes a critical aspect of balance control (Gera et al., 2018). It is believed that communication between the different sensory systems is lost in pathologic conditions such as, for example, in moderate/severe traumatic brain injury, cerebellar atrophy, and ataxia, causing moderate to severe postural instability (Guskiewicz, 2003). A concussion may lead to dysfunction of neurotransmission (Solar et al., 2024) and therefore may impair the brain’s ability to efficiently integrate the sensory inputs, especially during challenging surface conditions (e.g., unstable surface conditions). A study applying magnetoencephalography in a sample of military members with a history of repetitive subconcussive impacts revealed disrupted neuronal activity, including neural slowing, and functional dysconnectivity (Solar et al., 2024).

Here, the application of fNIRS above the motor-sensory cortex showed over all postural control conditions that concussed athletes with ongoing symptoms are characterized by increased brain oxygenation, particularly within the parietal cortex. This is not the case in asymptomatic and/or control athletes. The balance control system involves a hierarchical network of neural centers such as the cerebral cortex, cerebellum, basal ganglia, brainstem, and spinal cord that are related by peripheral and central feedback mechanisms and controlling voluntary movements (Guskiewicz, 2003). Higher level (/hierarchical) systems of postural control include the basal ganglia, cerebral cortex, and cerebellum, whereas lower levels are represented by the brainstem and spinal cord (Lalonde & Strazielle, 2007). Whereas lower hierarchical regions trigger more automatic postural responses, the cerebral cortex is involved in postural control each time a person must consciously maintain balance, for example, while walking across a slippery floor (Kandel et al., 2013). The present data showed that individuals of all experimental groups increase their brain processes when balancing either with closed eyes (vs. open eyes) or when controlling posture during unstable (vs. stable) surface conditions. Increased brain activation overall balance conditions in symptomatic concussed athletes (but not in asymptomatic and control athletes), therefore, indicates that these individuals activate higher hierarchal neuronal resources to control for postural sway. It has been argued that concussed individuals may increase attention and cognitive effort when compared to individuals without a history of concussion (Jacob et al., 2022). Because the cerebral cortex has more control over anticipatory postural adjustments than automatic postural reactions (Kandel et al., 2013), the present findings indicate that postural control does not constitute an automatized process but rather relies on explicit cortical control in individuals that suffer from long-term post-concussion symptoms.

Multisensory integration showed to be impaired in individuals with lesions to the parietal cortex (Derouesne et al., 1984) and can increase body sway (Pérennou et al., 2000;Young et al., 2020). Here, increased brain oxygenation patterns were not only evident in concussed and symptomatic athletes within the parietal cortex but also overall individuals during eyes closed (vs. eyes open) conditions as well as during unstable (vs. stable) surface conditions. This not only evidences that the parietal cortex serves sensorimotor integration when receiving input from lower body movements, and movements coordinating multiple body parts (Sereno & Huang, 2014) but also points out that it may constitute a (I) characteristic brain region of sensory integration during postural control tasks as well as (II) a specific region of post-concussion balance deficits. Higher activation within the parietal cortex was also observed in a previous study applying fNIRS during postural control in a youth group with recent concussions (Urban et al., 2020). It has been suggested that the additional recruitment of certain brain regions following a concussion may reflect increased mental processing required to perform a task (Bryer et al., 2013;Plenger et al., 2016;Urban et al., 2020). Thus, the present findings indicate that concussed and symptomatic athletes must not only explicitly control posture but also the parietal cortex upregulates its activity for additional sensory integration processes post-concussion.

Previous studies that investigated skilled athletes showed that such individuals were characterized by decreased of cortical activity during visuo-motor performances (Del Percio et al., 2009). In fact, skilled athletes with high postural control showed to recruit brain cortices for additional cognitive tasks other than balance control (Chen et al., 2023). These findings are in line with the “neural efficiency hypothesis” indicating that highly trained skills become more automated (Debarnot et al., 2014) and therefore necessitate minimal energy consumption (Nakata & Yabe, 2001). The fact that individuals with post-concussion symptoms are characterized by increased brain activation within parietal cortices overall postural control conditions therefore indicates that these individuals upregulate sensory integration processing more than healthy individuals do in order to keep balance under control. However, as the sway data indicate, even increased parietal activity does not help to counteract existing balance deficits. Thus, the overactivation of such athletes may constitute an ineffective way to compensate for postural deficits as such processes may serve a compensatory function (Reuter-Lorenz & Cappell, 2008). We, therefore, conclude that balance control does not constitute an automatized process in concussed athletes that suffer from post-concussion symptoms but the upregulation of neural activation within the parietal cortex indicates an inefficient (/compensatory) neural mechanism to integrate additional sensory information for deficient balance control.

### 4.3 Limitations

While we report new findings about neural and behavioral characteristics of athletes with long-term post-concussion symptoms, it is important to acknowledge the limitations of this study. Our sample consisted of unbalanced groups with regards to gender. Knowing that there may exist gender differences regarding clinical symptoms, alterations in brain structure and function, and recovery trajectories (Koerte et al., 2020), future studies must account for balanced groups to better understand potential outcomes between the sexes.

We also did not account for the heterogeneity of injury mechanisms, locations, and severity. Furthermore, both concussed groups reported their (multiple) concussive injuries long-term after the incident. Because previous studies applying balance protocols and fNIRS analyses focused on youth athletes only with experienced concussions within weeks of injury (e.g.,Urban et al., 2020), different results may be grounded in the different samples. Thus, next steps must not only consider the investigation of athletes before and after concussions but also add investigations about short- and long-term post-concussion parameters within the same sample.

Regarding the method of analysis, fNIRS is an increasingly used method in neuroscience research. However, it is also limited in its application. When compared to fMRI, it lacks spatial sensitivity as it is restricted to superficial layers of the cortex. Furthermore, whereas a typical voxel size in human fMRI detects signals within the range of millimeters, fNIRS detects signals only within centimeters. Similar to fMRI, fNIRS relies on neurovascular coupling to infer changes in neuronal activity. However, there is the possibility that changes of oxygenation may not uniquely be caused by neurovascular coupling. Change in fNIRS signals can also be due to changes in intracerebral hemodynamics caused by task-related systemic activity and/or extracerebral hemodynamics (Tachtsidis & Scholkmann, 2016). By including short-separation channels (to regress out the superficial hemodynamics) and carefully designing the balance protocol (e.g., several blocks per condition; not comparing versus baselines but between conditions), we tried to minimize potential “false positives” within the present study.

## 5 Conclusion

This study provides new evidence about the neuronal and behavioral outcomes of long-term post-concussion symptoms in athletes. The combined application of postural control tasks and fNIRS above motor-sensory cortices showed that concussed athletes with postural deficits are characterized by increased brain oxygenation, particularly within the parietal cortex. Such neural upregulation indicates that postural control in concussed athletes constitutes a rather conscious than unconscious process. Individuals without post-concussion symptoms may control posture by a more automatic/unconscious strategy. Thus, concussed and symptomatic athletes increase their cognitive effort to control posture, which may increase energy demands. Because it has been argued that higher susceptibility to injuries after concussion may be grounded in impaired motor-sensory control mechanisms (Chmielewski et al., 2021), we conclude that upregulation of parietal activity during sensory integration processes constitutes a characteristic of concussed individuals. Whether this pattern is related to an increased risk of injury must be investigated in future studies.

## Figures and Tables

**Appendix Fig. A2.**
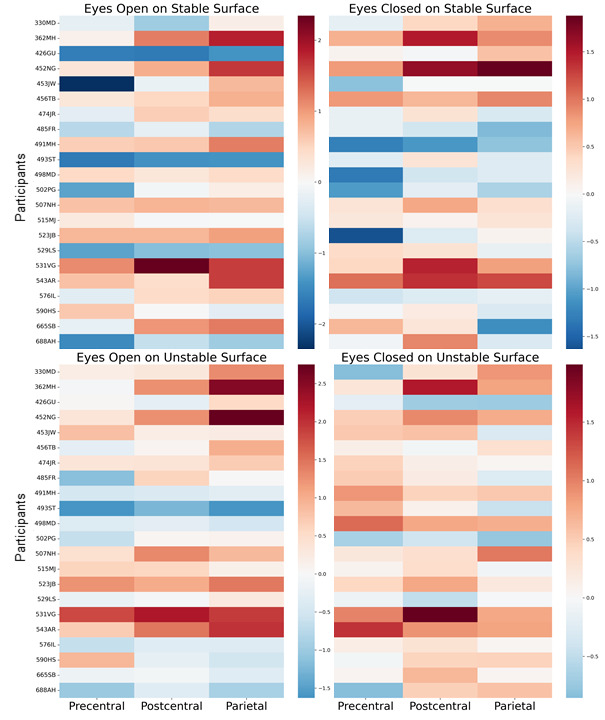
Individual brain oxygenation variation maps (∆HbO_2_) of concussed and symptomatic versus concussed and asymptomatic athletes within precentral, postcentral, and parietal brain regions during (4) postural control conditions (with eyes open/closed and during stable/unstable surface conditions).

## Data Availability

The data will be shared upon request.

## References

[IMAG.a.50-b1] Al-Husseini , A. , Gard , A. , Fransson , P.-A. , Tegner , Y. , Magnusson , M. , Marklund , N. , & Tjernström , F. ( 2022 ). Long-term postural control in elite athletes following mild traumatic brain injury . Frontiers in Neurology , 13 , 906594 . 10.3389/fneur.2022.906594 36172026 PMC9511028

[IMAG.a.50-b5] Broglio , S. P. , & Puetz , T. W. ( 2008 ). The effect of sport concussion on neurocognitive function, self-report symptoms and postural control . Sports Medicine , 38 ( 1 ), 53 – 67 . 10.2165/00007256-200838010-00005 18081367

[IMAG.a.50-b6] Brooks , M. A. , Peterson , K. , Biese , K. , Sanfilippo , J. , Heiderscheit , B. C. , & Bell , D. R. ( 2016 ). Concussion increases odds of sustaining a lower extremity musculoskeletal injury after return to play among collegiate athletes . The American Journal of Sports Medicine , 44 ( 3 ), 742 – 747 . 10.1177/0363546515622387 26786903

[IMAG.a.50-b7] Bryer , E. J. , Medaglia , J. D. , Rostami , S. , & Hillary , F. G. ( 2013 ). Neural recruitment after mild traumatic brain injury is task dependent: A meta-analysis . Journal of the International Neuropsychological Society: JINS , 19 ( 7 ), 751 – 762 . 10.1017/S1355617713000490 23656706

[IMAG.a.50-b8] Buckley , T. , Murray , N. G. , Munkasy , B. A. , Oldham , J. R. , Evans , K. M. , & Clouse , B. ( 2021 ). Impairments in dynamic postural control across concussion clinical milestones . Journal of Neurotrauma , 38 ( 1 ), 86 – 93 . 10.1089/neu.2019.6910 32674657 PMC7757537

[IMAG.a.50-b10] Caccese , J. B. , Santos , F. V. , Yamaguchi , F. K. , Buckley , T. A. , & Jeka , J. J. ( 2021 ). Persistent visual and vestibular impairments for postural control following concussion: A cross-sectional study in university students . Sports Medicine , 51 ( 10 ), 2209 – 2220 . 10.1007/s40279-021-01472-3 33881749 PMC8449812

[IMAG.a.50-b11] Cavanaugh , J. T. , Guskiewicz , K. M. , Giuliani , C. , Marshall , S. , Mercer , V. , & Stergiou , N. ( 2005 ). Detecting altered postural control after cerebral concussion in athletes with normal postural stability . British Journal of Sports Medicine , 39 ( 11 ), 805 – 811 . 10.1136/bjsm.2004.015909 16244188 PMC1725054

[IMAG.a.50-b12] Chen , J. , Kwok , A. P. K. , & Li , Y. ( 2023 ). Effective utilization of attentional resources in postural control in athletes of skill-oriented sports: An event-related potential study . Frontiers in Human Neuroscience , 17 , 1219022 . 10.3389/FNHUM.2023.1219022/BIBTEX 37694171 PMC10483146

[IMAG.a.50-b13] Chmielewski , T. L. , Tatman , J. , Suzuki , S. , Horodyski , M. B. , Reisman , D. S. , Bauer , R. M. , Clugston , J. R. , & Herman , D. C. ( 2021 ). Impaired motor control after sport-related concussion could increase risk for musculoskeletal injury: Implications for clinical management and rehabilitation . Journal of Sport and Health Science , 10 ( 2 ), 154 – 161 . 10.1016/J.JSHS.2020.11.005 33188963 PMC7987572

[IMAG.a.50-b14] Daneshvar , D. H. , Mez , J. , Alosco , M. L. , Baucom , Z. H. , Mahar , I. , Baugh , C. M. , Valle , J. P. , Weuve , J. , Paganoni , S. , Cantu , R. C. , Zafonte , R. D. , Stern , R. A. , Stein , T. D. , Tripodis , Y. , Nowinski , C. J. , & McKee , A. C. ( 2021 ). Incidence of and mortality from amyotrophic lateral sclerosis in national football league athletes . JAMA Network Open , 4 ( 12 ), 2138801 . 10.1001/JAMANETWORKOPEN.2021.38801 PMC8674746 34910152

[IMAG.a.50-b15] Debarnot , U. , Sperduti , M. , Di Rienzo , F. , & Guillot , A. ( 2014 ). Experts bodies, experts minds: How physical and mental training shape the brain . Frontiers in Human Neuroscience , 8 , 280 . 10.3389/FNHUM.2014.00280 24847236 PMC4019873

[IMAG.a.50-b16] De Beaumont , L. , Brisson , B. , Lassonde , M. , & Jolicoeur , P. ( 2007 ). Long-term electrophysiological changes in athletes with a history of multiple concussions . Brain Injury , 21 ( 6 ), 631 – 644 . 10.1080/02699050701426931 17577714

[IMAG.a.50-b200] De Beaumont , L. , Lassonde , M. , Leclerc , S. , & Théoret , H . ( 2007 ). Long-term and cumulative effects of sports concussion on motor cortex inhibition . Neurosurgery , 61 ( 2 ), 329 – 337 . 10.1227/01.NEU.0000280000.03578.B6 17762745

[IMAG.a.50-b17] De Beaumont , L. , Mongeon , D. , Tremblay , S. , Messier , J. , Prince , F. , Leclerc , S. , Lassonde , M. , & Théoret , H. ( 2011 ). Persistent motor system abnormalities in formerly concussed athletes . Journal of Athletic Training , 46 ( 3 ), 234 – 240 . 10.4085/1062-6050-46.3.234 21669091 PMC3419550

[IMAG.a.50-b18] De Beaumont , L. , Tremblay , S. , Poirier , J. , Lassonde , M. , & Théoret , H. ( 2012 ). Altered bidirectional plasticity and reduced implicit motor learning in concussed athletes . Cerebral Cortex (New York, N.Y.: 1991) , 22 ( 1 ), 112 – 121 . 10.1093/CERCOR/BHR096 21572090

[IMAG.a.50-b19] Del Percio , C. , Babiloni , C. , Marzano , N. , Iacoboni , M. , Infarinato , F. , Vecchio , F. , Lizio , R. , Aschieri , P. , Fiore , A. , Toràn , G. , Gallamini , M. , Baratto , M. , & Eusebi , F. ( 2009 ). “ Neural efficiency” of athletes’ brain for upright standing: A high-resolution EEG study . Brain Research Bulletin , 79 ( 3–4 ), 193 – 200 . 10.1016/J.BRAINRESBULL.2009.02.001 19429191

[IMAG.a.50-b20] Derouesne , C. , Mas , J. L. , Bolgert , F. , & Castaigne , P. ( 1984 ). Pure sensory stroke caused by a small cortical infarct in the middle cerebral artery territory . Stroke , 15 ( 4 ), 660 – 662 . 10.1161/01.STR.15.4.660 6464057

[IMAG.a.50-b21] Didehbani , N. , Cullum , C. M. , Mansinghani , S. , Conover , H. , & Hart , J. ( 2013 ). Depressive symptoms and concussions in aging retired NFL players . Archives of Clinical Neuropsychology , 28 ( 5 ), 418 – 424 . 10.1093/arclin/act028 23644673 PMC4007104

[IMAG.a.50-b23] Eagle , S. R. , Kontos , A. P. , Pepping , G. J. , Johnson , C. D. , Sinnott , A. , LaGoy , A. , & Connaboy , C. ( 2020 ). Increased risk of musculoskeletal injury following sport-related concussion: A perception–action coupling approach . Sports Medicine , 50 ( 1 ), 15 – 23 . 10.1007/S40279-019-01144-3 31228023

[IMAG.a.50-b24] Echemendia , R. J. , Brett , B. L. , Broglio , S. , Davis , G. A. , Giza , C. C. , Guskiewicz , K. M. , Harmon , K. G. , Herring , S. , Howell , D. R. , Master , C. L. , Valovich McLeod , T. C. , McCrea , M. , Naidu , D. , Patricios , J. , Putukian , M. , Walton , S. R. , Schneider , K. J. , Burma , J. S. , & Bruce , J. M. ( 2023 ). Introducing the Sport Concussion Assessment Tool 6 (SCAT6) . British Journal of Sports Medicine , 57 ( 11 ), 619 – 621 . 10.1136/BJSPORTS-2023-106849 37316207

[IMAG.a.50-b28] Felipe , L. ( 2021 ). Concussion and balance in sports . In R. Taiar (Ed.), *Contemporary advances in sports science* . IntechOpen . 10.5772/intechopen.97024

[IMAG.a.50-b29] Fino , P. C. , Becker , L. N. , Fino , N. F. , Griesemer , B. , Goforth , M. , & Brolinson , P. G. ( 2019 ). Effects of recent concussion and injury history on instantaneous relative risk of lower extremity injury in division I collegiate athletes . Clinical Journal of Sport Medicine: Official Journal of the Canadian Academy of Sport Medicine , 29 ( 3 ), 218 – 223 . 10.1097/JSM.0000000000000502 31033615

[IMAG.a.50-b201] Fishburn , F. A. , Ludlum , R. S. , Vaidya , C. J. , & Medvedev , A. V. ( 2019 ). Temporal Derivative Distribution Repair (TDDR): A motion correction method for fNIRS . NeuroImage , 184 , 171 – 179 . 10.1016/J.NEUROIMAGE.2018.09.025 30217544 PMC6230489

[IMAG.a.50-b30] Freedman , D. J. , & Ibos , G. ( 2018 ). An integrative framework for sensory, motor, and cognitive functions of posterior parietal cortex . Neuron , 97 ( 6 ), 1219 . 10.1016/J.NEURON.2018.01.044 29566792 PMC5884170

[IMAG.a.50-b31] Gagnon , L. , Perdue , K. , Greve , D. , Goldenholz , D. , Kaskhedikar , G. , & Boas , D. ( 2011 ). Improved recovery of the hemodynamic response in diffuse optical imaging using short optode separations and state-space modeling . NeuroImage , 56 ( 3 ), 1362 – 1371 . 10.1016/J.NEUROIMAGE.2011.03.001 21385616 PMC3085546

[IMAG.a.50-b32] Gera , G. , Chesnutt , J. , Mancini , M. , Horak , F. B. , & King , L. A. ( 2018 ). Inertial sensor-based assessment of central sensory integration for balance after mild traumatic brain injury . Military Medicine , 183 ( Suppl. 1 ), 327 – 332 . 10.1093/milmed/usx162 PMC6501206 29635623

[IMAG.a.50-b33] Geurts , A. C. H. , Ribbers , G. M. , Knoop , J. A. , & Van Limbeek , J. ( 1996 ). Identification of static and dynamic postural instability following traumatic brain injury . Archives of Physical Medicine and Rehabilitation , 77 ( 7 ), 639 – 644 . 10.1016/S0003-9993(96)90001-5 8669988

[IMAG.a.50-b34] Giza , C. C. , & Hovda , D. A. ( 2014 ). The new neurometabolic cascade of concussion . Neurosurgery , 75 , 24 – 33 . 10.1227/NEU.0000000000000505 PMC4479139 25232881

[IMAG.a.50-b35] Guskiewicz , K. M. ( 2003 ). Assessment of postural stability following sport-related concussion . Current Sports Medicine Reports , 2 ( 1 ), 24 – 30 . https://doi.org/10.1249/00149619-200302000-00006 12831673 10.1249/00149619-200302000-00006

[IMAG.a.50-b36] Guskiewicz , K. M. ( 2011 ). Balance assessment in the management of sport-related concussion . Clinics in Sports Medicine , 30 ( 1 ), 89 – 102 . 10.1016/j.csm.2010.09.004 21074084

[IMAG.a.50-b38] Guskiewicz , K. M. , Riemann , B. L. , Perrin , D. H. , & Nashner , L. M. ( 1997 ). Alternative approaches to the assessment of mild head injury in athletes . Medicine and Science in Sports and Exercise , 29 ( 7 Suppl. ), 213 – 221 . 10.1097/00005768-199707001-00003 9247918

[IMAG.a.50-b39] Guskiewicz , K. M. , Ross , S. E. , & Marshall , S. W. ( 2001 ). Postural stability and neuropsychological deficits after concussion in collegiate athletes . Journal of Athletic Training , 36 ( 3 ), 263 – 273 . 10.1136/bjsm.2004.015909 12937495 PMC155417

[IMAG.a.50-b41] Helmich , I. , Berger , A. , & Lausberg , H. ( 2016 ). Neural control of posture in individuals with persisting postconcussion symptoms . Medicine & Science in Sports & Exercise , 48 ( 12 ), 2362 – 2369 . 10.1249/MSS.0000000000001028 27387294

[IMAG.a.50-b43] Helmich , I. , Coenen , J. , Henckert , S. , Pardalis , E. , Schupp , S. , & Lausberg , H. ( 2020 ). Reduced frontopolar brain activation characterizes concussed athletes with balance deficits . NeuroImage: Clinical , 25 , 102164 . 10.1016/j.nicl.2020.102164 31954336 PMC6965737

[IMAG.a.50-b44] Herman , D. C. , Jones , D. , Harrison , A. , Moser , M. , Tillman , S. , Farmer , K. , Pass , A. , Clugston , J. R. , Hernandez , J. , & Chmielewski , T. L. ( 2017 ). Concussion may increase the risk of subsequent lower extremity musculoskeletal injury in collegiate athletes . Sports Medicine (Auckland, N.Z.) , 47 ( 5 ), 1003 – 1010 . 10.1007/S40279-016-0607-9 27544666 PMC5318296

[IMAG.a.50-b45] Hocke , L. M. , Duszynski , C. C. , Debert , C. T. , Dleikan , D. , & Dunn , J. F. ( 2018 ). Reduced functional connectivity in adults with persistent post-concussion symptoms—A functional near-infrared spectroscopy study . Journal of Neurotrauma , 35 ( 11 ), 1224 – 1232 . 10.1089/neu.2017.5365 29373947 PMC5962910

[IMAG.a.50-b46] Horak , F. B. ( 2006 ). Postural orientation and equilibrium: What do we need to know about neural control of balance to prevent falls? Age and Ageing , 35 ( Suppl. 2 ), ii7 – ii11 . 10.1093/ageing/afl077 16926210

[IMAG.a.50-b47] Howell , D. R. , Buckley , T. A. , Lynall , R. C. , & Meehan , W. P. ( 2018 ). Worsening dual-task gait costs after concussion and their association with subsequent sport-related injury . Journal of Neurotrauma , 35 ( 14 ), 1630 – 1636 . 10.1089/NEU.2017.5570 29490564

[IMAG.a.50-b48] Howell , D. R. , Lynall , R. C. , Buckley , T. A. , & Herman , D. C. ( 2018 ). Neuromuscular control deficits and the risk of subsequent injury after a concussion: A scoping review . Sports Medicine , 48 ( 5 ), 1097 – 1115 . 10.1007/S40279-018-0871-Y 29453743 PMC6037312

[IMAG.a.50-b49] Ingersoll , C. D. , & Armstrong , C. W. ( 1992 ). The effects of closed-head injury on postural sway . Medicine & Science in Sports & Exercise , 24 ( 7 ), 739 – 743 . 10.1249/00005768-199207000-00001 1501556

[IMAG.a.50-b51] Jacob , D. , Unnsteinsdóttir Kristensen , I. S . , Aubonnet , R. , Recenti , M. , Donisi , L. , Ricciardi , C. , Svansson , H. Á. R. , Agnarsdóttir , S. , Colacino , A. , Jónsdóttir , M. K. , Kristjánsdóttir , H. , Sigurjónsdóttir , H. Á. , Cesarelli , M. , Eggertsdóttir Claessen , L. Ó. , Hassan , M. , Petersen , H. , & Gargiulo , P. ( 2022 ). Towards defining biomarkers to evaluate concussions using virtual reality and a moving platform (Biovrsea) . Scientific Reports , 12 ( 1 ), 8996 . 10.1038/s41598-022-12822-0 35637235 PMC9151646

[IMAG.a.50-b202] Jasper , H. ( 1958 ). Report of the committee on methods and clinical examination in electroencephalography . Electroencephalography and Clinical Neurophysiology , 10 , 371 – 375 . 10.1016/0013-4694(58)90053-1

[IMAG.a.50-b52] Johnston , W. , Coughlan , G. F. , & Caulfield , B. ( 2017 ). Challenging concussed athletes: The future of balance assessment in concussion . QJM , 110 ( 12 ), 779 – 783 . 10.1093/qjmed/hcw228 28040709

[IMAG.a.50-b53] Joubran , K. , Bar-Haim , S. , & Shmuelof , L. ( 2022 ). The functional and structural neural correlates of dynamic balance impairment and recovery in persons with acquired brain injury . Scientific Reports , 12 ( 1 ), 1 – 11 . 10.1038/s41598-022-12123-6 35568728 PMC9107482

[IMAG.a.50-b54] Kahya , M. , Moon , S. , Ranchet , M. , Vukas , R. R. , Lyons , K. E. , Pahwa , R. , Akinwuntan , A. , & Devos , H. ( 2019 ). Brain activity during dual task gait and balance in aging and age-related neurodegenerative conditions: A systematic review . Experimental Gerontology , 128 , 110756 . 10.1016/J.EXGER.2019.110756 31648005 PMC6876748

[IMAG.a.50-b55] Kandel , E. R. , Schwartz , J. H. , Jessel , T. M. , Siegelbaum , S. A. , & Hudspeth , A. J. ( 2013 ). Principles of neural sciences , McGraw-Hill Medical . https://catalog.nlm.nih.gov/permalink/01NLM_INST/1o1phhn/alma9915857043406676

[IMAG.a.50-b56] Kaulmann , D. , Hermsdörfer , J. , & Johannsen , L. ( 2017 ). Disruption of right posterior parietal cortex by continuous Theta Burst Stimulation alters the control of body balance in quiet stance . The European Journal of Neuroscience , 45 ( 5 ), 671 – 678 . 10.1111/EJN.13522 28092413

[IMAG.a.50-b57] Klautke , J. , Foster , C. , Medendorp , W. P. , & Heed , T. ( 2023 ). Dynamic spatial coding in parietal cortex mediates tactile-motor transformation . Nature Communications , 14 ( 1 ), 1 – 18 . 10.1038/s41467-023-39959-4 PMC10374589 37500625

[IMAG.a.50-b58] Kleffelgaard , I. , Roe , C. , Soberg , H. L. , & Bergland , A. ( 2012 ). Associations among self-reported balance problems, post-concussion symptoms and performance-based tests: A longitudinal follow-up study . Disability and Rehabilitation , 34 ( 9 ), 788 – 794 . 10.3109/09638288.2011.619624 22149161

[IMAG.a.50-b61] Koerte , I. K. , Schultz , V. , Sydnor , V. J. , Howell , D. R. , Guenette , J. P. , Dennis , E. , Kochsiek , J. , Kaufmann , D. , Sollmann , N. , Mondello , S. , Shenton , M. E. , & Lin , A. P. ( 2020 ). Sex-related differences in the effects of sports-related concussion: A review . Journal of Neuroimaging: Official Journal of the American Society of Neuroimaging , 30 ( 4 ), 387 . 10.1111/JON.12726 32533752 PMC8221087

[IMAG.a.50-b63] Kumai , K. , Ikeda , Y. , Sakai , K. , Goto , K. , Morikawa , K. , & Shibata , K. ( 2022 ). Brain and muscle activation patterns during postural control affect static postural control . Gait & Posture , 96 , 102 – 108 . 10.1016/j.gaitpost.2022.05.017 35635985

[IMAG.a.50-b64] Lalonde , R. , & Strazielle , C. ( 2007 ). Brain regions and genes affecting postural control . Progress in Neurobiology , 81 ( 1 ), 45 – 60 . 10.1016/J.PNEUROBIO.2006.11.005 17222959

[IMAG.a.50-b65] Langlois , J. A. , Rutland-Brown , W. , & Wald , M. M. ( 2006 ). The epidemiology and impact of traumatic brain injury: A brief overview . Journal of Head Trauma Rehabilitation , 21 ( 5 ), 375 – 378 . 10.1097/00001199-200609000-00001 16983222

[IMAG.a.50-b66] Lehman , E. J. , Hein , M. J. , Baron , S. L. , & Gersic , C. M. ( 2012 ). Neurodegenerative causes of death among retired National Football League players . Neurology , 79 ( 19 ), 1970 . 10.1212/WNL.0B013E31826DAF50 22955124 PMC4098841

[IMAG.a.50-b67] Lin , L. F. , Liou , T.-H. , Hu , C.-J. , Ma , H.-P. , Ou , J.-C. , Chiang , Y.-H. , Chiu , W.-T. , Tsai , S.-H. , & Chu , W.-C. ( 2015 ). Balance function and sensory integration after mild traumatic brain injury . Brain Injury , 29 ( 1 ), 41 – 46 . 10.3109/02699052.2014.955881 25265292

[IMAG.a.50-b68] Lovell , M. R. , Iverson , G. L. , Collins , M. W. , Podell , K. , Johnston , K. M. , Pardini , D. , Pardini , J. E. , Norwig , J. , & Maroon , J. C. ( 2006 ). Measurement of symptoms following sports-related concussion: Reliability and normative data for the post-concussion scale . Applied Neuropsychology , 13 ( 3 ), 166 – 174 . 10.1207/s15324826an1303 17361669

[IMAG.a.50-b203] Lührs , M. , & Goebel , R. ( 2017 ). Turbo-Satori: A neurofeedback and brain-computer interface toolbox for real-time functional near-infrared spectroscopy . Neurophotonics , 4 ( 4 ), 1 . 10.1117/1.NPH.4.4.041504 PMC5629919 29021985

[IMAG.a.50-b69] Lynall , R. C. , Mauntel , T. C. , Padua , D. A. , & Mihalik , J. P. ( 2015 ). Acute lower extremity injury rates increase after concussion in college athletes . Medicine and Science in Sports and Exercise , 47 ( 12 ), 2487 – 2492 . 10.1249/MSS.0000000000000716 26057941

[IMAG.a.50-b70] Mackay , D. F. , Russell , E. R. , Stewart , K. , MacLean , J. A. , Pell , J. P. , & Stewart , W. ( 2019 ). Neurodegenerative disease mortality among former professional soccer players . New England Journal of Medicine , 381 ( 19 ), 1801 – 1808 . 10.1056/NEJMOA1908483/SUPPL_FILE/NEJMOA1908483_DISCLOSURES.PDF 31633894 PMC8747032

[IMAG.a.50-b71] Manley , G. , Gardner , A. J. , Schneider , K. J. , Guskiewicz , K. M. , Bailes , J. , Cantu , R. C. , Castellani , R. J. , Turner , M. , Jordan , B. D. , Randolph , C. , Dvořák , J. , Hayden , K. A. , Tator , C. H. , McCrory , P. , & Iverson , G. L. ( 2017 ). A systematic review of potential long-term effects of sport-related concussion . British Journal of Sports Medicine , 51 ( 12 ), 969 – 977 . 10.1136/bjsports-2017-097791 28455362 PMC5466926

[IMAG.a.50-b72] Martini , D. N. , Gera , G. , Brumbach , B. H. , Campbell , K. R. , Parrington , L. , Chesnutt , J. , & King , L. A. ( 2023 ). Symptoms and central sensory integration in people with chronic mTBI: Clinical implications . Military Medicine , 188 ( 11–12 ), 3553 – 3560 . 10.1093/milmed/usac157 35657326 PMC10629982

[IMAG.a.50-b75] McCrory , P. , Meeuwisse , W. , Dvorak , J. , Aubry , M. , Bailes , J. , Broglio , S. , Cantu , R. C. , Cassidy , D. , Echemendia , R. J. , Castellani , R. J. , Davis , G. A. , Ellenbogen , R. , Emery , C. , Engebretsen , L. , Feddermann-Demont , N. , Giza , C. C. , Guskiewicz , K. M. , Herring , S. , Iverson , G. L. ,… Vos , P. E. ( 2017 ). Consensus statement on concussion in sport—The 5 ^th^ international conference on concussion in sport held in Berlin, October 2016 . British Journal of Sports Medicine , 51 ( 11 ), 838 – 847 . 10.1136/bjsports-2017-097699 28446457

[IMAG.a.50-b76] McPherson , A. L. , Nagai , T. , Webster , K. E. , & Hewett , T. E. ( 2019 ). Musculoskeletal injury risk after sport-related concussion: A systematic review and meta-analysis . American Journal of Sports Medicine , 47 ( 7 ), 1754 – 1762 . 10.1177/0363546518785901/ASSET/IMAGES/LARGE/10.1177_0363546518785901-FIG3.JPEG 30074832

[IMAG.a.50-b79] Nakata , H. , & Yabe , K. ( 2001 ). Automatic postural response systems in individuals with congenital total blindness . Gait & Posture , 14 ( 1 ), 36 – 43 . 10.1016/S0966-6362(00)00100-4 11378423

[IMAG.a.50-b80] Nguyen , V. T. , Zafonte , R. D. , Chen , J. T. , Kponee-Shovein , K. Z. , Paganoni , S. , Pascual-Leone , A. , Speizer , F. E. , Baggish , A. L. , Taylor , H. A. , Nadler , L. M. , Courtney , T. K. , Connor , A. , & Weisskopf , M. G. ( 2019 ). Mortality among professional american-style football players and professional American baseball players . JAMA Network Open , 2 ( 5 ), e194223 . 10.1001/JAMANETWORKOPEN.2019.4223 31125098 PMC6632140

[IMAG.a.50-b83] Patricios , J. S. , Schneider , K. J. , Dvorak , J. , Ahmed , O. H. , Blauwet , C. , Cantu , R. C. , Davis , G. A. , Echemendia , R. J. , Makdissi , M. , Mcnamee , M. , Broglio , S. , Emery , C. A. , Kutcher , J. S. , Leddy , J. J. , Maddocks , D. , Manley , G. , Mccrea , M. , Purcell , L. K. , Putukian , M. ,… Meeuwisse , W. ( 2023 ). Consensus statement on concussion in sport: The 6th International Conference on Concussion in Sport-Amsterdam, October 2022 . British Journal of Sports Medicine , 57 , 695 – 711 . 10.1136/bjsports-2023-106898 37316210

[IMAG.a.50-b84] Pérennou , D. A. , Leblond , C. , Amblard , B. , Micallef , J. P. , Rouget , E. , & Pélissier , J. ( 2000 ). The polymodal sensory cortex is crucial for controlling lateral postural stability: Evidence from stroke patients . Brain Research Bulletin , 53 ( 3 ), 359 – 365 . 10.1016/S0361-9230(00)00360-9 11113593

[IMAG.a.50-b85] Peterka , R. J. ( 2002 ). Sensorimotor integration in human postural control . Journal of Neurophysiology , 88 ( 3 ), 1097 – 1118 . 10.1152/jn.2002.88.3.1097 12205132

[IMAG.a.50-b86] Peterka , R. J. ( 2018 ). Sensory integration for human balance control . In Handbook of clinical neurology (Vol. 159 , pp. 27 – 42 ). Elsevier . 10.1016/B978-0-444-63916-5.00002-1 30482320

[IMAG.a.50-b87] Plenger , P. , Krishnan , K. , Cloud , M. , Bosworth , C. , Qualls , D. , & Marquez de la Plata , C. ( 2016 ). fNIRS-based investigation of the Stroop task after TBI . Brain Imaging and Behavior , 10 ( 2 ), 357 – 366 . 10.1007/S11682-015-9401-9 26058665

[IMAG.a.50-b88] Powers , K. C. , Cinelli , M. E. , & Kalmar , J. M. ( 2014 ). Cortical hypoexcitability persists beyond the symptomatic phase of a concussion . Brain Injury , 28 ( 4 ), 465 – 471 . 10.3109/02699052.2014.888759 24702432

[IMAG.a.50-b89] Purkayastha , S. , Adair , H. , Woodruff , A. , Ryan , L. J. , Williams , B. , James , E. , & Bell , K. R. ( 2019 ). Balance testing following concussion: Postural sway versus complexity index . PM&R , 11 ( 11 ), 1184 – 1192 . 10.1002/pmrj.12129 30729729

[IMAG.a.50-b90] Reuter-Lorenz , P. A. , & Cappell , K. A. ( 2008 ). Neurocognitive aging and the compensation hypothesis . Current Directions in Psychological Science , 17 ( 3 ), 177 – 182 . 10.1111/J.1467-8721.2008.00570.X/ASSET/IMAGES/LARGE/10.1111_J.1467-8721.2008.00570.X-FIG2.JPEG

[IMAG.a.50-b95] Schmidt , J. D. , Terry , D. P. , Ko , J. , Newell , K. M. , & Miller , L. S. ( 2018 ). Balance regularity among former high school football players with or without a history of concussion . Journal of Athletic Training , 53 ( 2 ), 109 – 114 . 10.4085/1062-6050-326-16 29332469 PMC5842900

[IMAG.a.50-b204] Sereno , M. I. , & Huang , R. S . ( 2014 ). Multisensory maps in parietal cortex . Current Opinion in Neurobiology , 24 ( 1 ), 39 – 46 . 10.1016/J.CONB.2013.08.014 24492077 PMC3969294

[IMAG.a.50-b205] Shumway-Cook , A. , & Horak , F. B. ( 1986 ). Assessing the influence of sensory interaction on balance: Suggestion from the field . Physical Therapy , 66 ( 10 ), 1548 – 1550 . 10.1093/ptj/66.10.1548 3763708

[IMAG.a.50-b97] Slobounov , S. , Sebastianelli , W. , & Moss , R. ( 2005 ). Alteration of posture-related cortical potentials in mild traumatic brain injury . Neuroscience Letters , 383 ( 3 ), 251 – 255 . 10.1016/j.neulet.2005.04.039 15876490

[IMAG.a.50-b98] Solar , K. G. , Ventresca , M. , Zamyadi , R. , Zhang , J. , Jetly , R. , Vartanian , O. , Rhind , S. G. , & Dunkley , B. T. ( 2024 ). Repetitive subconcussion results in disrupted neural activity independent of concussion history . Brain Communications , 6 ( 5 ), fcae348 . 10.1093/BRAINCOMMS/FCAE348 39440300 PMC11495223

[IMAG.a.50-b99] Solis-Escalante , T. , Van Der Cruijsen , J. , De Kam , D. , Van Kordelaar , J. , Weerdesteyn , V. , & Schouten , A. C. ( 2019 ). Cortical dynamics during preparation and execution of reactive balance responses with distinct postural demands . NeuroImage , 188 , 557 – 571 . 10.1016/j.neuroimage.2018.12.045 30590120

[IMAG.a.50-b100] Sosnoff , J. J. , Broglio , S. P. , Shin , S. , & Ferrara , M. S. ( 2011 ). Previous mild traumatic brain injury and postural-control dynamics . Journal of Athletic Training , 46 ( 1 ), 85 – 91 . 10.4085/1062-6050-46.1.85 21214355 PMC3017494

[IMAG.a.50-b101] Stangl , M. , Maoz , S. L. , & Suthana , N. ( 2023 ). Mobile cognition: Imaging the human brain in the “real world. ” Nature Reviews Neuroscience , 24 , 347 – 362 . 10.1038/s41583-023-00692-y 37046077 PMC10642288

[IMAG.a.50-b103] Tachtsidis , I. , & Scholkmann , F. ( 2016 ). False positives and false negatives in functional near-infrared spectroscopy: Issues, challenges, and the way forward . Neurophotonics , 3 ( 3 ), 031405 . 10.1117/1.NPH.3.3.031405 27054143 PMC4791590

[IMAG.a.50-b104] Thompson , J. , Sebastianelli , W. , & Slobounov , S. ( 2005 ). EEG and postural correlates of mild traumatic brain injury in athletes . Neuroscience Letters , 377 ( 3 ), 158 – 163 . 10.1016/j.neulet.2004.11.090 15755518

[IMAG.a.50-b106] Ueda , P. , Pasternak , B. , Lim , C.-E. , Neovius , M. , Kader , M. , Forssblad , M. , Ludvigsson , J. F. , & Svanström , H. ( 2023 ). Neurodegenerative disease among male elite football (soccer) players in Sweden: A cohort study . The Lancet Public Health , 8 ( 4 ), e256 – e265 . 10.1016/s2468-2667(23)00027-0 36934741

[IMAG.a.50-b108] Urban , K. , Schudlo , L. , Keightley , M. , Alain , S. , Reed , N. , & Chau , T. ( 2020 ). Altered brain activation in youth following concussion: Using a dual-task paradigm . Developmental Neurorehabilitation , 24 ( 3 ), 187 – 198 . 10.1080/17518423.2020.1825539 33012188

[IMAG.a.50-b109] Valovich McLeod , T. C. , & Hale , T. D. ( 2015 ). Vestibular and balance issues following sport-related concussion . Brain Injury , 29 ( 2 ), 175 – 184 . 10.3109/02699052.2014.965206 25291297

[IMAG.a.50-b110] Van Der Kooij , H. , & Peterka , R. J. ( 2011 ). Non-linear stimulus-response behavior of the human stance control system is predicted by optimization of a system with sensory and motor noise . Journal of Computational Neuroscience , 30 ( 3 ), 759 – 778 . 10.1007/s10827-010-0291-y 21161357 PMC3108015

[IMAG.a.50-b112] Wilkerson , G. B. , Grooms , D. R. , & Acocello , S. N. ( 2017 ). Neuromechanical considerations for postconcussion musculoskeletal injury risk management . Current Sports Medicine Reports , 16 ( 6 ), 419 – 427 . 10.1249/JSR.0000000000000430 29135640

[IMAG.a.50-b114] Wood , T. A. , Hsieh , K. L. , An , R. , Ballard , R. A. , & Sosnoff , J. J. ( 2019 ). Balance and gait alterations observed more than 2 weeks after concussion . American Journal of Physical Medicine & Rehabilitation , 98 ( 7 ), 566 – 576 . 10.1097/PHM.0000000000001152 31219809

[IMAG.a.50-b117] Young , D. R. , Parikh , P. J. , & Layne , C. S. ( 2020 ). Non-invasive brain stimulation of the posterior parietal cortex alters postural adaptation . Frontiers in Human Neuroscience , 14 , 248 . 10.3389/FNHUM.2020.00248 32676017 PMC7333640

[IMAG.a.50-b119] Yücel , M. A. , Lühmann , A. v. , Scholkmann , F. , Gervain , J. , Dan , I. , Ayaz , H. , Boas , D. , Cooper , R. J. , Culver , J. , Elwell , C. E. , Eggebrecht , A. , Franceschini , M. A. , Grova , C. , Homae , F. , Lesage , F. , Obrig , H. , Tachtsidis , I. , Tak , S. , Tong , Y. ,… Wolf , M. ( 2021 ). Best practices for fNIRS publications . Neurophotonics , 8 ( 1 ), 012101 . 10.1117/1.NPH.8.1.012101 33442557 PMC7793571

[IMAG.a.50-b120] Yücel , M. A. , Selb , J. J. , Huppert , T. J. , Franceschini , M. A. , & Boas , D. A. ( 2017 ). Functional near infrared spectroscopy: Enabling routine functional brain imaging . Current Opinion in Biomedical Engineering , 4 , 78 – 86 . 10.1016/J.COBME.2017.09.011 29457144 PMC5810962

[IMAG.a.50-b121] Zimeo Morais , G. A. , Balardin , J. B. , & Sato , J. R. ( 2018 ). fNIRS Optodes’ Location Decider (fOLD): A toolbox for probe arrangement guided by brain regions-of-interest . Scientific Reports , 8 ( 1 ), 1 – 11 . 10.1038/s41598-018-21716-z 29463928 PMC5820343

